# Hydrochlorothiazide Use and Risk of Nonmelanoma Skin Cancers: A Biological Plausibility Study

**DOI:** 10.1155/2021/6655542

**Published:** 2021-08-14

**Authors:** Elisabetta Bigagli, Lorenzo Cinci, Mario D'Ambrosio, Patrizia Nardini, Francesca Portelli, Roberta Colucci, Maura Lodovici, Alessandro Mugelli, Cristina Luceri

**Affiliations:** ^1^Department of Neuroscience, Psychology, Drug Research and Child Health (NEUROFARBA), Section of Pharmacology and Toxicology, University of Florence, Florence, Italy; ^2^Department of Clinical and Experimental Medicine, Section of Histology, University of Florence, Florence, Italy; ^3^Department of Health Sciences, Section of Anatomical Pathology, Careggi University Hospital, Florence, Italy; ^4^Department of Health Sciences, Section of Dermatology, University of Florence, Florence, Italy

## Abstract

Recent studies reported the association between increased risk of nonmelanoma skin cancers (NMSCs) and the use of hydrochlorothiazide (HCTZ), one of the most commonly prescribed diuretic, antihypertensive drug, over the world. Although HCTZ is known to be photosensitizing, the mechanisms involved in its potential prophotocarcinogenic effects remain unclear. Under acute exposure, therapeutically relevant concentrations of HCTZ (70, 140, and 370 ng/mL) amplified UVA-induced double-strand breaks, oxidative DNA, and protein damage in HaCaT human keratinocytes, and this effect was associated to a defective activity of the DNA repair enzyme, OGG1. Oxidative damage to DNA, but not that to proteins, was reversible within few hours. After chronic, combined exposure to HCTZ (70 ng/mL) and UVA (10 J/cm^2^), for 9 weeks, keratinocytes acquired a dysplastic-like phenotype characterized by a multilayered morphology and alterations in cell size, shape, and contacts. At the ultrastructural level, several atypical and enlarged nuclei and evident nucleoli were also observed. These transformed keratinocytes were apoptosis resistant, exhibited enhanced clonogenicity capacity, increased DNA damage and inflammation, defective DNA repair ability, and increased expression of the oncogene *Δ*Np63*α* and intranuclear *β*-catenin accumulation (a hallmark of Wnt pathway activation), compared to those treated with UVA alone. None of these molecular, morphological, or functional effects were observed in cells treated with HCTZ alone. All these features resemble in part those of preneoplastic lesions and NMSCs and provide evidence of a biological plausibility for the association among exposure to UVA, use of HCTZ, and increased risk of NMSCs. These results are of translational relevance since we used environmentally relevant UVA doses and tested HCTZ at concentrations that reflect the plasma levels of doses used in clinical practice. This study also highlights that drug safety data should be followed by experimental evaluations to clarify the mechanistic aspects of adverse events.

## 1. Introduction

Recent pharmacoepidemiological studies showed a cumulative, dose-dependent association between hydrochlorothiazide (HCTZ), one of the most widely prescribed diuretic, antihypertensive drug, over the world [1], and increased nonmelanoma skin cancers (NMSCs) risk [2, 3]. On these bases, the European Medicine Agency Pharmacovigilance Risk Assessment Committee, recommended to amend the Summary of Product Characteristics for HCTZ containing products with special warnings and precautions [4]. Thereafter, data from the World Health Organization pharmacovigilance database VigiBase and case–control studies confirmed this association [5–9]. Very recently, the Food and Drug Administration also approved changes to the HCTZ drug label to inform patients about this risk (https://www.fda.gov/drugs/drug-safety-and-availability/fda-approves-label-changes-hydrochlorothiazide-describe-small-risk-non-melanoma-skin-cancer#:~:text=%5B8%2F20%2F2020%5D,HCTZ%20use%20and%20to%20encourage).

The most important risk factor for NMSC development is a constant and cumulative UVR exposure [10, 11] which is responsible for known harmful cellular effects consisting in a direct UVB-mediated DNA damage and in an indirect UVA-mediated oxidative DNA damage [12–14].

On the contrary, the molecular pathways at the basis of the increased risk of NMSCs observed among HCTZ users are unclear, but photosensitizing actions of HCTZ may play a role. Skin photosensitivity reactions, frequently associated to UVA exposure, were indeed reported in patients taking HCTZ [15–17]. A combined exposure to UVA and HCTZ induced acute phototoxicity in experimental studies in keratinocytes [18], in cervical cancer cells [19] and in hairless mice [20]. HCTZ also enhanced UVA-induced DNA damage in a mice model defective in nucleotide excision repair [21]. These data contributed to the decision by the International Agency for Research on Cancer Monograph Working Group, to classify HCTZ as possibly carcinogenic to humans (Group 2B) [22].

However, these studies were focused on acute effects and did not investigate the biological pathways linking the photosensitizing/phototoxic properties of HCTZ to its potential prophotocarcinogenic effects.

Since NMSCs (and among them, squamous cell carcinoma) arise from chronic, repeated UV exposures [10, 11, 23, 24], and HCTZ is mostly a long-term treatment, an experimental model involving long-lasting drug exposures and repeated UV irradiations might be a more reliable tool to explore the biological plausibility of the increased risk of NMSC among HCTZ users.

On these basis, we characterized both the acute and the long-term, molecular, morphological, and functional consequences of the combined exposure to HCTZ and UVA in human keratinocytes.

## 2. Materials and Methods

### 2.1. Cell Cultures

Human keratinocytes (HaCaT cells, American Type Culture Collection (Manassas, VA, USA) were cultured in Dulbecco's Modified Eagle's Medium (Lonza, Basel, Switzerland), supplemented with 10% fetal bovine serum, l-glutamine (2 mM), 100 unit/mL penicillin, and 100 *μ*g/mL streptomycin and maintained at 37°C in a humidified atmosphere containing 5% CO_2_. The cell line was authenticated by BMR Genomics (Padova, Italy) before manuscript submission.

### 2.2. Short-Term Treatments and Photoirradiation

**Figure 1:**
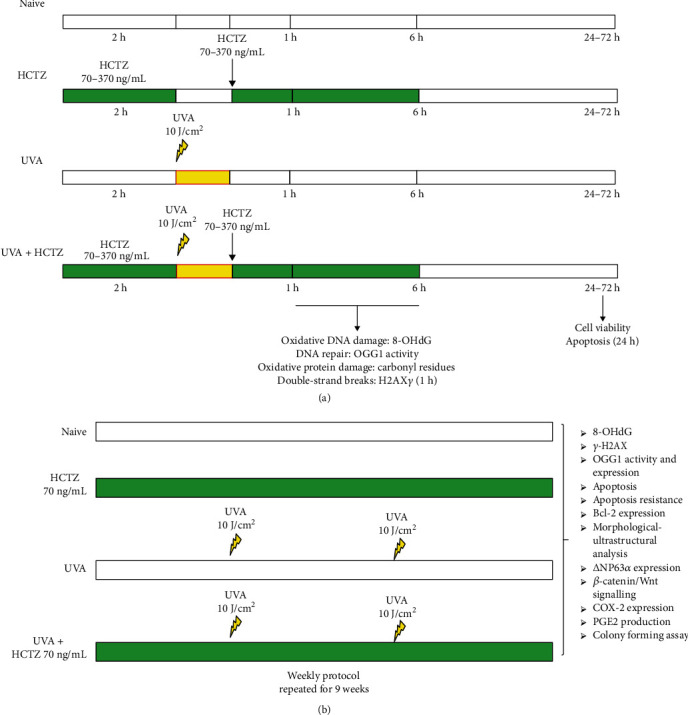
(a) Short-term treatments and photoirradiation: experimental flow chart. Cells were treated with HCTZ alone (70-370 ng/mL) or in combination with UVA (10 J/cm^2^), left untreated (naive), or irradiated with UVA only. 8-Hydroxy-2′-deoxyguanosine (8-OHdG), DNA repair (OGG1 activity), and carbonyl residues were determined after 1 and 6 h. *γ*-H2AX (double-strand breaks) was measured after 1 h. Cell viability was measured at 24, 48, and 72 h. Apoptosis was measured at 24 h. (b) Long-term treatments and photoirradiation are as follows: experimental flow chart of the weekly protocol. Cells were treated with HCTZ alone (70 ng/mL) or in combination with UVA (10 J/cm^2^) for 9 weeks, left untreated (naive), or irradiated with UVA only. 8-OHdG, OGG1 activity, genotoxic damage (*γ*-H2AX), apoptosis resistance, COX-2 and *Δ*Np63*α* expression, PGE2 production, and *β*-catenin localization were investigated together with morphological characterization and clonogenic capacity.

The experimental flow chart for short-term experiments is depicted in Figure 1(a). Control cells (naive) were maintained under standard culture condition without any treatment. In the HCTZ group, cells were treated with HCTZ alone at concentrations ranging from 70 to 370 ng/mL corresponding to the values of Cmax following the administration in humans of 12.5, 25, and 75 mg doses, respectively [25]. HCTZ (PubChem CID: 3639) (Sigma Aldrich, Milan, Italy) was dissolved in dimethyl sulfoxide (DMSO) as 14 mg/mL stock solutions and used immediately or stored at −20°C. In the UVA group, cells were irradiated with 10 J/cm^2^ of UVA light with an emission centered at 365 nm, using a UV Bio-Link (BLX) (Vilber Lourmat, Marne-La-Vallee, France), on cold plates to eliminate UVA-induced thermal effects and without lid. The time of irradiation lasted in 40 minutes. Before irradiating the cells with UVA, the medium was removed, and the cells were covered with a thin layer of phosphate buffer solution (PBS). In the HCTZ+UVA group, cells were preincubated with HCTZ (70-370 ng/mL) for 2 hours (h), exposed to 10 J/cm^2^ of UVA, and then treated with HCTZ for additional 1 or 6 h.

### 2.3. Long-Term Treatments and Photoirradiation

The experimental flow chart summarizing the weekly protocol for long-term experiments is depicted in Figure 1(b). For all the experiments conducted in cells chronically treated, 8 × 10^5^ cells, corresponding to 14,000 cells/cm^2^, were seeded in Petri dishes, allowed to grow for 24 h, and then treated as follows: cells maintained in standard culture conditions (naive); cells chronically treated with HCTZ alone (70 ng/mL) for 9 weeks (HCTZ); and cells irradiated with UVA alone (10 J/cm^2^), twice a week, for 9 weeks (UVA) or in the presence of HCTZ (70 ng/mL) (HCTZ +UVA). Each single exposure lasted for 40 minutes. Fresh HCTZ was added every 2 days. The concentration of 70 ng/mL is similar to that found at the steady state following the administration of the dose of 75 mg of HCTZ in humans [26], and the weekly UVA dose corresponds to a recreational human exposure of approximately 1 hour to midsummer sun [27].

### 2.4. Photocytotoxicity Assay

HaCaT cells (5 × 10^3^ cells/well corresponding to 10,000 cells/cm^2^) were seeded and after 24 h exposed to HCTZ (70-370 ng/mL) with or without UVA irradiation (10 J/cm^2^). Cytotoxicity was evaluated after 24, 48, and 72 h by using the MTS reagent (CellTiter 96 Aqueous Proliferation Assay; Promega Madison, WI, USA) as previously described [28].

### 2.5. Oxidative Protein Damage: Carbonyl Residues

HaCaT cells (2 × 10^5^ cells/well corresponding to 50,000 cells/cm^2^) were seeded and after 24 h exposed to HCTZ (70-370 ng/mL) with or without UVA irradiation (10 J/cm^2^). Carbonyl residues were determined following the method by Correa-Salde and Albesa [29] with few modifications as described in Bigagli et al. [30]. Protein content was estimated by using the Bio-Rad DC protein assay kit (Bio-Rad, Milan, Italy).

### 2.6. Oxidative DNA Damage: 8-OHdG Determination

HaCaT cells (5 × 10^5^ cells/well corresponding to 55,000 cells/cm^2^) were seeded and after 24 h exposed to HCTZ (70-370 ng/mL) with or without UVA irradiation (10 J/cm^2^). Total DNA was extracted using the DNeasy Mini Kit (Qiagen, Hilden, Germany), denatured at 95°C for 5 minutes, and digested with nuclease P1 and alkaline phosphatase at 37°C [31]. 8-OHdG levels were quantified using the 8-OHdG ELISA kit (Jaica, Fukuroi, Japan). The results were calculated from the absorbance at 450 nm of a standard curve of 8-OHdG solutions and expressed as ng of 8-OHdG/mL.

### 2.7. DNA Repair: OGG1 Activity

HaCaT cells (2 × 10^5^ cells/well corresponding to 50,000 cells/cm^2^) were seeded and after 24 h exposed to HCTZ (70-370 ng/mL) with or without UVA irradiation (10 J/cm^2^). The activity of OGG1 was determined by a protocol set-up in our laboratory on the basis of Hamann and Schwerdtle [32] with the following modifications: a hairpin-like structured synthetic oligonucleotide with the sequence.

5′CAATAATAACACGCXCGACCAGTCCTGCTTTTGCAGGACTGGTCGCGCGTGTTATTATTG-3′, *X* = 8 − OHdG, was designed by us and synthetized by Integrated DNA Technologies (Leuven, Belgium). Total protein (1 *μ*g) or 2 units of recombinant hOGG1, used as positive control (New England Biolabs, MA, USA), were incubated in a reaction mixture containing 1X NEBuffer 2, 40 pmol of synthetic oligonucleotide, and 100 *μ*g/mL BSA, at 37°C for 30 min and then at 95°C for 5 minutes to stop the reaction. A negative control, containing only the reaction mixture was also used. Samples were analyzed by electrophoresis in 3% agarose gel, in TBE 0.5X. The image of residual and intact (negative control) bands was acquired and analyzed through the software Quantity One (Bio-Rad). The percentage of residual oligo was calculated on the basis of the following formula: intact oligonucleotide : 100 = cleaved oligonucleotide : *x*. The OGG1 glycosylase activity was calculated as follows: 100–*x*.

### 2.8. Reverse Transcription Polymerase Chain Reaction (RT-PCR)

HaCaT cells (3 × 10^5^ cells/well corresponding to 75,000 cells/cm^2^) were seeded and after 24 h exposed to HCTZ (70-370 ng/mL) with or without UVA irradiation (10 J/cm^2^). RNA was extracted with TRIzol (Invitrogen, Carlsbad, USA) and reverse-transcribed using the RevertAid RT kit (Thermo Scientific, Whaltam, USA). Primers were designed on the basis of the human GenBank sequences, and GAPDH was coamplified as the reference [30]: GAPDH F: CCCTCAAGGGCATCCTGGGCT, R: GCAGGGACTCCCCAGCAGTGA; *Δ*NP63*α* F: TGCCCAGACTCAATTTAGTGAG, R: TCTGGATGGGGCATGTCTTTGC; OGG1, F: TATCGTGCCCGTTACGTGAG R: CACTGAACAGCACCGCTTG; COX-2 F: TTGCCCGACTCCCTTGGGTGT, R: CCTCCTGCCCCACAGCAAACC; and APE1 F: AGATCTCGCGAGTAGGGCAAC, R: TCCGAAGGAGCTGACCAGTA.

### 2.9. Prostaglandin E2 (PGE2) Determination

HaCaT cells (2 × 10^5^ cells/well corresponding to 50,000 cells/cm^2^) were seeded and after 24 h exposed to HCTZ (70-370 ng/mL) with or without UVA irradiation (10 J/cm^2^). PGE2 levels were measured in the supernatants using a commercial kit (Cayman, Ann Arbor, USA) according to the manufacturer's specifications.

### 2.10. Immunocytochemistry

HaCaT cells (2 × 10^5^ cells/well corresponding to 50,000 cells/cm^2^) were seeded. For the determination of *γ*-H2AX foci, serum starved, HaCaT cells at confluence, were used. Cells were fixed in 4% paraformaldehyde for 15 minutes, dehydrated in alcohol and paraffin embedded. Immunochemistry was performed on histological slices (4 *μ*m thick) as described by Cinci et al. [28]; primary antibodies are as follows: polyclonal mouse anti *β*-catenin (1 : 100; BD Bioscience, USA); polyclonal rabbit anti Bcl-2 (1 : 200; Cell Signaling, Danvers, USA); and phosphohistone H2A.X (Ser 139) rabbit antibody (1 : 50; Cell Signaling, Danvers, USA); and secondary antibodies are as follows: Alexa Fluor 488 goat anti-mouse or Alexa Fluor 594 goat anti-rabbit (Invitrogen, USA) 1.200. The analysis of *γ*-H2AX foci was performed after 1 h since this was previously identified as the time for maximal foci induction in HaCaT cells [33]. Cells were judged as positive for *γ*-H2AX foci if they displayed more than 10 discrete dots of brightness [34].

### 2.11. Apoptosis

For acute experiments, HaCaT cells were seeded on histological slide (2 × 10^5^ cells/well corresponding to 50,000 cells/cm^2^) and treated with HCTZ at the final concentration of 70, 140, and 370 ng/mL. After two hours, cells were irradiated with UVA (10 J/cm^2^) in 100 *μ*L of PBS. After irradiation, PBS was replaced with standard culture medium and incubated at 37°C for 24 h. Cells were washed in PBS and fixed in Bouin's liquid (acetic acid/formaldehyde/picric acid), for 20 minutes, and then stained with Feulgen's reaction, specific for DNA detection. Briefly, cells were incubated in 1 N HCL for 22 minutes at 60°C, with the Schiff reactive for 60 minutes at room temperature and then washed in 0.05 N HCL and 5% NaHSO_3_. Successively, nuclei were counterstained for 30 seconds with 0.5% fast green in alcoholic solution, dehydrated in ethanol, washed in xylene, and mounted [35].

For long-term experiments, cells chronically exposed to UVA and HCTZ, as described above, were harvested, fixed in Bouin's liquid for 18 h, and then paraffin embedded. Histological sections, 5 *μ*m thick, were obtained and stained with Feulgen's reaction, as described above.

For each sample, 10 images at ×1000 magnification were obtained. Apoptotic cells were recognized by the presence of two morphological prodromal features of apoptotic bodies: nuclear fragmentation and cellular limits evanescence. The percentage of apoptotic cells was carried out on five microscopic fields for each experimental condition with an average number of about 50 cells and was determined by two independent observers in a blind fashion.

### 2.12. Image Acquisition and Analysis

Microscopic analyses were performed with a fluorescence microscopy (Labophot-2, Nikon, Japan) connected to a CCD camera. Ten photomicrographs were randomly taken for each sample at ×400 magnification. The measurements were made by two independent, blinded investigators, using the ImageJ 1.33 image analysis software (https://rsb.info.nih.gov/ij).

### 2.13. Cytology

Cells were fixed in cold 4% paraformaldehyde for 15 minutes, dehydrated in alcohol, and then paraffin embedded. Sections (4 *μ*m thick) were stained with hematoxylin-eosin for morphological analysis.

### 2.14. Transmission Electron Microscopy (TEM)

To highlight cellular shape and contacts, glutaraldehyde 4% (Electron Microscopy Sciences, Hatfield, USA) in 0.1 mol/L cacodylate buffer, pH 7.4, was directly added to the cultures for 15 minutes at RT: then, the cells were scraped, harvested by centrifugation at 3000 rpm for 10 minutes, and maintained in fixative overnight. The cells were postfixed in osmium tetroxide (1%), dehydrated, and embedded in epoxy resin. Sections (70 nm thick) were examined by a JEOL 1010 transmission electron microscope (Jeol, Tokyo, Japan) at 80 kV, after contrast staining with uranyl acetate and lead citrate.

### 2.15. Resistance to Doxorubicin-Induced Apoptosis

Cells (5 × 10^3^ cells/well corresponding to 10,000 cells/cm^2^) were treated with doxorubicin (Sigma Aldrich, Milan, Italy) (10^−4^; 5 × 10^−5^; 10^−5^; 5 × 10^−6^; 10^−6^; 5 × 10^−7^; 10^−7^; 5 × 10^−8^; and 10^−8^ M) for 72 h. Cytotoxicity was evaluated by using the MTS reagent as previously described [28].

### 2.16. Anchorage-Independent Growth: The Soft Agar Colony Formation Assay

Anchorage-independent growth assay was performed as described by Borowicz et al. [36]. Briefly, in six-well plates, 2 mL agar medium (0.5% agar-agar in DMEM with 20% FBS) was added to each well and allowed to solidify. HaCaT cells (4 × 10^4^ cell/well corresponding to 4500 cells/cm^2^) were suspended in 1 mL DMEM with 20% FBS and 0.3% agar solution and then laid on top of the hardened agar medium. Cells were then incubated at 37°C in a 5% CO_2_ atmosphere for 21 days. Subsequently, the cells were stained with nitroblue tetrazolium chloride solution and incubated overnight at 37°C. Colonies were counted by two blinded independent observers.

### 2.17. Statistical Analysis

Results are presented as mean ± SEM of at least three independent experiments. Multiple comparisons were performed using 1-way ANOVA followed by Bonferroni's post hoc test. Difference with *p* < 0.05 was considered significant. Statistical analysis was performed with GraphPad Prism 5 (GraphPad software, San Diego, USA).

## 3. Results

### 3.1. Short-Term Exposure to HCTZ Enhances UVA-Induced DNA and Protein Damage and Decreases DNA Repair

**Figure 2:**
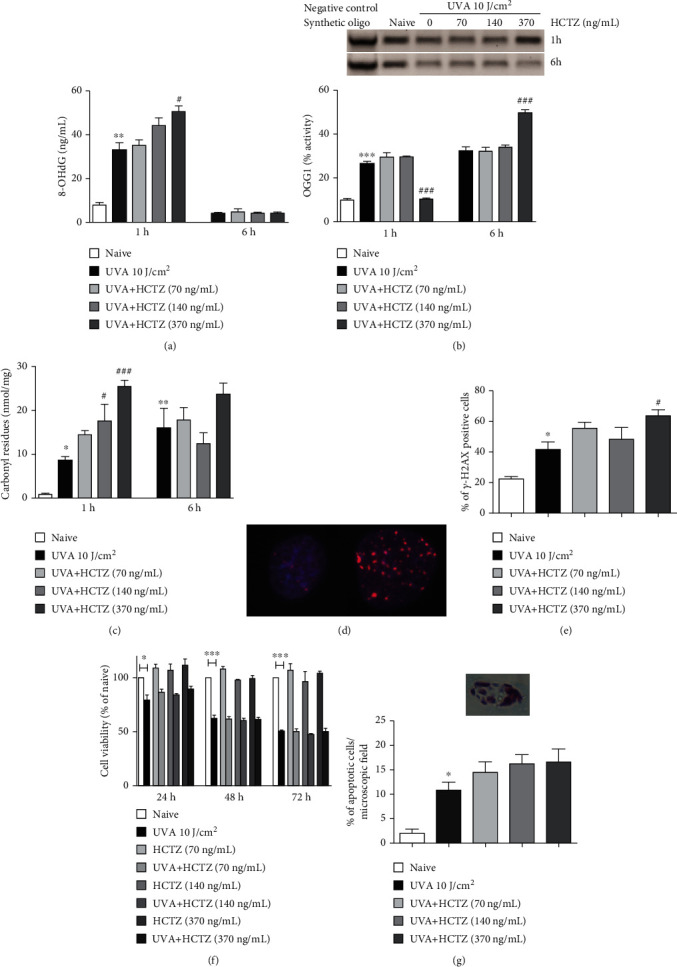
Short-term combined exposure to HCTZ and UVA increases oxidative DNA and protein damage and decreases the repair activity of OGG1. (a) 8-OHdG levels, OGG1 activity (b), and carbonyl residues (c) after 1 and 6 h. (b) representative image of the bands obtained by incubating 1 *μ*g of proteins with 40 pmol of the synthetic oligonucleotide containing 8-OHdG; the higher is the band intensity, the lower is OGG1 activity. (d) *γ*-H2AX immunostaining (red); nuclei were counterstained with DAPI (blue); the two pictures show a representative nucleus negative and positive for *γ*-H2AX immunofluorescence staining, respectively. (e) Quantification of the percentage of cells positive for *γ*-H2AX immunofluorescence staining. (f) Cell viability (% versus naive) measured at 24, 48, and 72 h. (g) Percentage of apoptotic cells measured at 24 h and a representative picture of an apoptotic cell showing prodromal features of apoptotic bodies: nuclear fragmentation and cellular limits evanescence. The values are expressed as means ± SEM (*n* = 3). ^∗^*p* < 0.05, ^∗∗^*p* < 0.01, and ^∗∗∗^*p* < 0.001 compared to naive; ^#^*p* < 0.05 and ^###^*p* < 0.001 compared to UVA alone. ANOVA with Bonferroni's posttest.

Within 1 h post irradiation, HCTZ increased the formation of 8-hydroxy-2′-deoxyguanosine (8-OHdG), compared to UVA alone (*p* < 0.01, posttest for linear trend), concentration-dependently, reaching up to 50% increase at 370 ng/mL (*p* < 0.05) (Figure 2(a)). At the same time point, in contrast to UVA radiation alone, the combined treatment with UVA and HCTZ (370 ng/mL) markedly reduced 8-oxoguanine DNA glycosylase 1 (OGG1) activity (by 60%) (*p* < 0.05) (Figure 2(b)). However, after 6 h, DNA damage was completely repaired (Figure 2(a)), and both the mRNA expression (1.52 ± 0.001 vs. 0.41 ± 0.08; *p* < 0.001) and OGG1 activity were significantly increased in cells treated with UVA and HCTZ (370 ng/mL) compared to UVA alone (*p* < 0.05) (Figure 2(b)).

Carbonyl residues were concentration-dependently enhanced by UVA+HCTZ at 1 h post irradiation (*p* < 0.05 at 140 ng/mL vs. UVA; *p* < 0.001 at 370 ng/mL vs. UVA), and their levels remained elevated also after 6 h (Figure 2(c)).

The percentage of cells positive for *γ*-H2AX, a marker for DNA double-strand breaks, was significantly increased in cells treated with UVA compared to naive (*p* < 0.05) and further enhanced in UVA+HCTZ compared to UVA alone (*p* < 0.01 at 370 ng/mL vs. UVA) (Figure 2(e)). The clear induction of distinct *γ*-H2AX foci in cells treated with UVA+HCTZ compared to control cells is shown in Figure 2(d).

UVA caused a time-dependent reduction in cell viability (-20% after 24 h, *p* < 0.05; -40% after 48 h, *p* < 0.001; and -50% after 72 h, *p* < 0.001). HCTZ alone was not cytotoxic nor did it exert additive cytotoxic effects in the presence of UVA (Figure 2(f)). Despite the trend toward increased apoptosis in the function of HCTZ concentration (*p* < 0.05, posttest for linear trend), no significant differences were observed compared to UVA alone (Figure 2(g)). A representative image of an apoptotic cell is shown in the inset of Figure 2(g).

### 3.2. Long-Term Combined Exposure to HCTZ and UVA Induces Dysplastic Features in Human Keratinocytes

**Figure 3:**
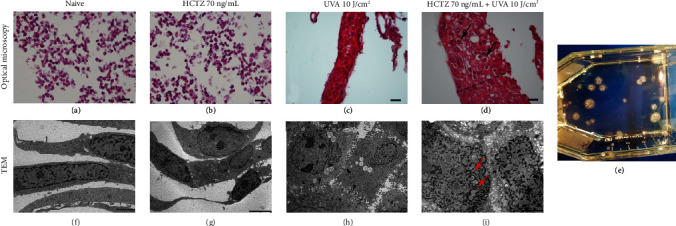
Long-term combined exposure to HCTZ and UVA induces dysplastic features in human keratinocytes. (a–d) Hematoxylin-eosin staining, sagittal sections. (d) Pseudoepithelial morphology with several cellular layers (black dashed line) and dysplastic features (enlarged nuclei and vacuolated cytoplasm black arrows) in UVA+HCTZ cells; magnification ×400, scale bar: 20 *μ*m. UVA+HCTZ colonies visible to the naked eye (e). (f–i) Transmission electron microscopy; naive and HCTZ cells (magnification ×10,000, scale bar 2 *μ*m (f); magnification ×5000, scale bar 5 *μ*m (g). Cytoplasmic bridges without direct cell to cell contacts (black arrow heads) in UVA cells (h) (magnification ×6000, scale bar 6 *μ*m); epithelial-like structure (black asterisk) with convoluted nuclei (red arrows) and a “salt and pepper” chromatin pattern in UVA+HCTZ (i) (magnification ×12,000, scale bar 2 *μ*m).

By the 7th week, keratinocytes treated with both UVA and HCTZ, but not those treated with HCTZ or UVA alone, started to grow as cellular aggregates and formed colonies visible to the naked eye (Figure 3(e)), exhibiting morphological and ultrastructural alterations suggestive of their transformation. In particular, after 9 weeks of treatment, cells treated with UVA alone or in combination with HCTZ (70 ng/mL) exhibited a pseudoepithelial morphology (Figures 3(c) and 3(d)) but only UVA+HCTZ cells, microscopically appeared as a multilayered pseudoepithelium (Figure 3(d), black dashed line) characterized by many cells with dysplastic features such as altered cytoplasmic to nuclear ratio, vacuolated cytoplasm, atypical and enlarged nuclei, and evident nucleoli (Figure 3(d), black arrows). At the ultrastructural level, UVA cells were polygonal in shape and displayed a number of cytoplasmic bridges without direct cell to cell contacts (Figure 3(h), black arrow heads); on the contrary, UVA+HCTZ cells were completely juxtaposed, mimicking an epithelial-like structure (Figure 3(i), black asterisk) with convoluted nuclei (red arrows) and a “salt and pepper” chromatin pattern (Figure 3(i)). Notably, cells treated with HCTZ alone (Figure 3(b)) displayed a round-shaped morphology identical to that of naive cells (Figure 3(a)) and grew in monolayer, and no ultrastructural abnormalities were noted (Figures 3(f) and 3(g)).

### 3.3. Long-Term Combined Exposure to HCTZ and UVA Increases Oxidative DNA Damage, Double-Strand Breaks, and Inflammation and Decreases DNA Repair

**Figure 4:**
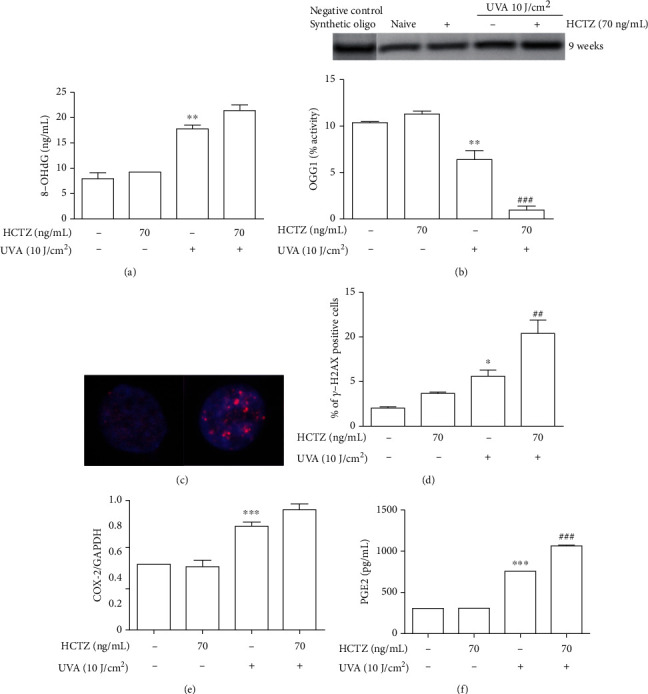
Long-term combined exposure to HCTZ and UVA increases oxidative DNA damage, genotoxicity, and inflammation and decreases the repair activity of OGG1. (a) 8-OHdG levels and (b) OGG1 activity. (c, d) *γ*-H2AX immunostaining (red); nuclei were counterstained with DAPI (blue); top insert in (c) shows the representative nuclei negative and positive for *γ*-H2AX immunofluorescence staining, respectively. (d) Quantification of the percentage of cells positive for *γ*-H2AX immunofluorescence staining. (e) COX-2 mRNA expression and (f) PGE2 levels. The values are expressed as means ± SEM (*n* = 3). ^∗^*p* < 0.05, ^∗∗^*p* < 0.01, and ^∗∗∗^*p* < 0.001 as compared to control cells (naive); ^###^*p* < 0.001 as compared to UVA alone; ANOVA test with Bonferroni's posttest.

Long-term treatment with UVA+HCTZ slightly increased the levels of 8-OHdG compared to UVA alone (Figure 4(a)) but drastically decreased OGG1 activity (-85%) (*p* < 0.001) (Figure 4(b)) and transcription (0.19 ± 0.01 vs. 0.78 ± 0.02; *p* < 0.001). In response to UVA alone or to UVA+HCTZ, the expression of APE1 was induced, but the difference was not statistically significant (0.53 ± 0.05 in naive compared to 0.89 ± 0.14 in UVA-treated cells and 1.06 ± 0.13 in UVA+HCTZ cells). The double-strand breaks marker *γ*-H2AX was significantly increased in cells treated with UVA compared to naive (*p* < 0.05) and further enhanced in UVA+HCTZ compared to UVA alone (*p* < 0.01) (Figures 4(c) and 4(d)). UVA+HCTZ-treated cells displayed a slight increase in COX-2 mRNA expression (Figure 4(e)) and significantly elevated production of prostaglandin E2 (PGE2) compared to UVA alone (Figure 4(f)) (*p* < 0.05). No effect of HCTZ alone was observed.

### 3.4. Long-Term Combined Exposure to HCTZ and UVA Increases *Δ*Np63*α* and Nuclear *β*-Catenin Expression and Confers Resistance to the Apoptogenic Effect of Doxorubicin

**Figure 5:**
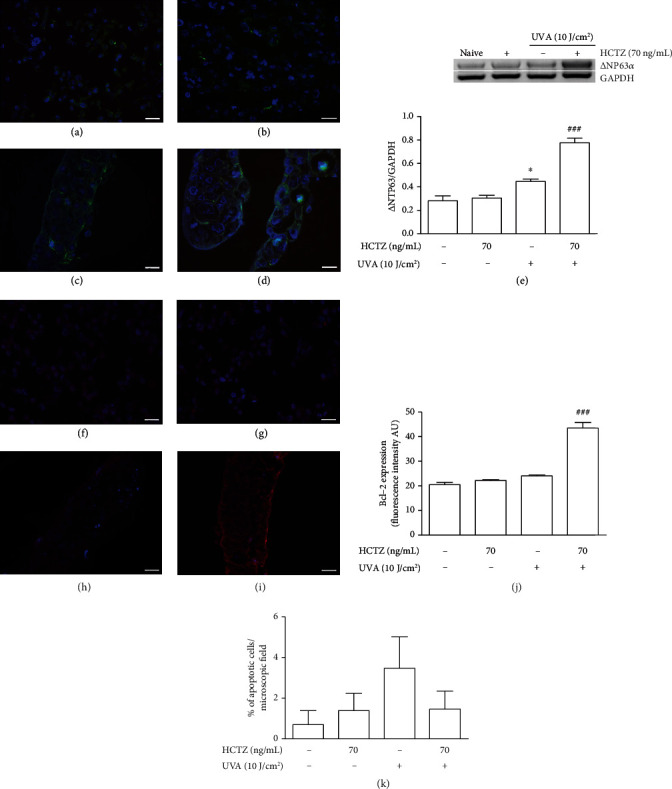
Long-term combined exposure to HCTZ and UVA activates *β*-catenin/Wnt oncogenic pathway, increases the expression of the oncogene and stem cell marker *Δ*Np63*α*, and upregulates the antiapoptotic protein Bcl-2. (a–d) Immunofluorescence staining for *β*-catenin expression (green); nuclei were counterstained with DAPI (blue); magnification ×400, scale bar 20 *μ*m. The top right insert in (d) shows the magnification of a representative cell with nuclear distribution of *β*-catenin. (e) *Δ*Np63*α* mRNA expression. (f–i) Immunofluorescence staining for Bcl-2 (red); nuclei were counterstained with DAPI (blue); magnification ×400, scale bar 20 *μ*m. (j) Quantification of the immunofluorescence staining for Bcl-2. (k) Percentage of apoptotic cells. The values are expressed as means ± SEM (*n* = 3). ^∗^*p* < 0.05 vs. control cells (naive); ^###^*p* < 0.001 as compared to UVA alone. The ANOVA test with Bonferroni's posttest.

Combined exposure to HCTZ and UVA significantly induced the accumulation of *β*-catenin in the nuclei, a marker of Wnt signaling activation, compared to UVA alone (11.8% ± 3.9 vs. 1.43% ± 0.71; *p* < 0.05, Figures 5(c) and 5(d)); conversely, in naive cells and in those treated with HCTZ alone, *β*-catenin expression was restricted to the cytoplasm (Figures 5(a) and 5(b)). The expression of *Δ*Np63*α* was higher in cells treated with UVA alone (*p* < 0.05 vs. naive), markedly enhanced in UVA+HCTZ cells (*p* < 0.001 vs. UVA) and similar to that of naive cells when HCTZ was administered alone (Figure 5(e)).

The percentage of apoptotic cells upon long-term treatment with either UVA alone (3.47%) or UVA+HCTZ (1.44%) (Figure 5(k)) was lower than that observed after acute exposure (UVA, 10.78%, and UVA+HCTZ 70 ng/mL, 14.45%) (Figure 2(g)). Qualitatively, we did not observe clear signs of necrosis such as ghosts (empty cellular membrane residues) or nuclear fragments without cytoplasmic residues. The expression of the antiapoptotic protein Bcl-2 was also significantly increased in UVA+HCTZ cells compared to UVA alone (*p* < 0.001; Figures 5(h)–5(j)) whereas no difference was observed between UVA alone and naive cells. To evaluate the acquisition of a generalized resistance to apoptosis, doxorubicin, a well-known proapoptogenic compound [37], was used and this assay showed that UVA+HCTZ cells were more resistant to doxorubicin than those treated with UVA alone as demonstrated by the increased IC50 (drug concentration required to inhibit cell growth by 50%). The IC50 value for doxorubicin was, in fact, 6-folds higher in UVA+HCTZ cells compared to UVA (30 ± 4.2 *μ*M vs. 4.5 ± 1.1 *μ*M, *p* < 0.05). Naive and cells treated with HCTZ alone showed similar IC50 values for doxorubicin, 0.8 ± 0.13 *μ*M and 0.7 ± 0.11 *μ*M, respectively.

### 3.5. Long-Term Combined Exposure to HCTZ and UVA Enhances the Clonogenicity Capacity

We used the soft agar colony formation assay, a well-known functional test based on the capability of transformed cells to grow independently of a solid surface and considered a hallmark of in vitro carcinogenesis [36].

**Figure 6:**
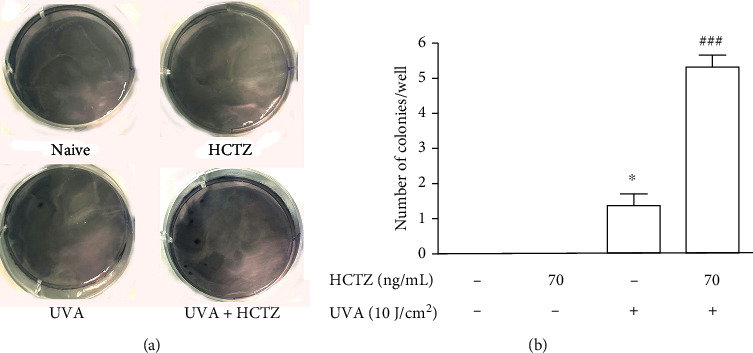
(a) Representative image of the anchorage-independent growth assay. (b) Quantification of the anchorage-independent growth assay. The values are expressed as means ± SEM (*n* = 3). ^∗^*p* < 0.05 vs. control cells (naive); ^###^*p* < 0.001 as compared to UVA alone. The ANOVA test with Bonferroni's posttest.

Naive and HaCaT cells treated with HCTZ alone did not form any colony in anchorage-independent (soft agar) clonogenicity assay. As expected for not fully transformed cells, on UVA+HCTZ-treated cells, we were able to detect only few colonies, but compared to UVA alone, they exhibited enhanced clonogenic potential, further suggesting their early-stage oncogenic transformation. On average, HaCaT cells treated with UVA alone formed only 1 colony/well, while the number of colonies formed by UVA+HCTZ cells was markedly enhanced compared to UVA alone (*p* < 0.001 vs. UVA, Figures 6(a) and 6(b)).

## 4. Discussion

**Figure 7:**
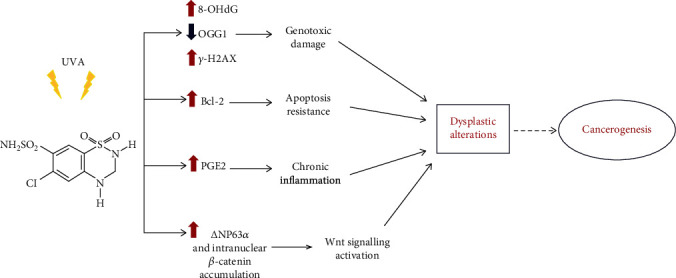
Schematic figure depicting the proposed mechanisms for the prophotocarcinogenic effects of HCTZ. Increased genotoxicity coupled with decreased ability to repair oxidative DNA damage and reduced susceptibility to apoptosis increases the likelihood of mutations and together with the activation of *Δ*Np63, and *β*-catenin/Wnt signaling may favor the early steps of squamous cell carcinoma development.

Our study demonstrates that human keratinocytes chronically coexposed with UVA and HCTZ, but not with HCTZ alone, develop a dysplastic morphology, and acquire molecular characteristics of an oncogenic transformation, thus providing biologically plausible mechanisms (Figure 7) for the increased risk of NMSCs observed in patients taking HCTZ [2, 3, 5–9].

HCTZ causes photosensitization by either type I (free radical) or type II (singlet molecular oxygen) mechanisms and undergoes photodehalogenation, yielding a reactive form, which can damage DNA, lipids, and proteins [38–40]. Seto et al. [41] also demonstrated that HCTZ is photoreactive and generates singlet oxygen.

As early as 1 h after acute exposure, HCTZ enhances the UVA-induced formation of 8-OHdG, a major mutagenic oxidative DNA lesion [42], and of *γ*-H2AX, a sensitive marker of double-strand breaks and photogenotoxicity [34, 43]. Double-strand breaks may derive from DNA replication or as a result of the repair process [44]; although we cannot completely exclude that some *γ*-H2AX foci were due to the replication process, they are more likely attributable to a direct DNA damage since we used serum-starved, confluent cells. Moreover, the presence of discrete *γ*-H2AX foci, rather than a pan-nuclear staining, supports a mechanism independent on repair [33, 45].

To ensure genomic integrity, 8-OHdG is quickly removed by OGG1; whether immediate or slightly delayed, the induction of OGG1 repair activity mitigates the accumulation of 8-OHdG, consistently with the high efficiency of base excision repair enzymes [14]. OGG1, as other repair proteins, may itself be susceptible to damage and inactivation by oxidation [46] that may persist longer than DNA damage since protein carbonyls are irreversible modifications that cannot be repaired and require proteasome degradation [47]. Although at the highest concentration of HCTZ tested, OGG1 activity was strongly abated because oxidation, an alternative defense mechanism involving its increased gene transcription, was put in place.

In a mice model defective in nucleotide excision repair, Kunisada et al. [21] demonstrated that a single dose of HCTZ enhanced UVA-induced cyclobutane pyrimidine dimers (CPDs), the most abundant form of DNA damage induced in human skin by UVA [48]. Although we cannot rule out the involvement of other oxidative lesions, the formation of CPDs and 8-OHdG generally exceed that of single-strand breaks and oxidized pyrimidines in a 10 : 3 : 1 : 1 ratio [49, 50].

At the molecular level, the presence of double-strand breaks and 8-OHdG lesions coupled with a defective DNA repair activity may lead to genomic mutations; this seems particularly relevant in the basal layer of human epidermis where keratinocytes express less OGG1 compared to the superficial layer [51, 52]. Moreover, there is evidence that OGG1 knockout mice are more susceptible to skin carcinogenesis [53], and both the loss of OGG1 and the accumulation of 8-OHdG have been observed in NMSCs [54]. However, there are also other repair proteins whose impairment may have consequences on the removal of damage: the nucleotide excision repair inhibition by UVA-photoactivated fluoroquinolones and vemurafenib has been reported [55] as well as oxidation of MYH and RPA proteins [46].

In addition to genomic insults, cotreatment with UVA and HCTZ activates Wnt, one of the oncogenic pathways involved in skin tumorigenesis [56], and increases inflammation, consistently with the upregulation of a number of proinflammatory cytokines observed in mice treated with HCTZ and UVB [57]. Indeed, human NMSCs and their precursors exhibit increased levels of PGE2 [58–60] and nuclear *β*-catenin localization [61, 62].

Human preneoplastic lesions and NMSCs also express *Δ*Np63*α* [63–66], an oncogene involved in the early steps of squamous cell carcinoma development [67] that may regulate or may be directly regulated by *β*-catenin [68, 69].

It is interesting to note that, after chronic treatment, the level of apoptosis was lower than that observed after acute treatment in cells treated with UVA in the presence of HCTZ, suggesting the acquisition of apoptosis resistance, also supported by the enhanced expression of *Δ*Np63*α* and Bcl-2. To explore whether chronic treatment with HCTZ and UVA caused the acquisition of resistance to proapoptotic insults, cells were treated with the known proapoptotic drug doxorubicin, observing a significant increase of its IC50 value. He et al. [37] reported that long-term exposure to UVA causes resistance to doxorubicin-induced apoptosis in HaCaT keratinocytes. Moreover, previous evidence demonstrated that the overexpression of *Δ*Np63*α* inhibited doxorubicin-induced apoptosis in HaCaT keratinocytes, independently of wild-type p53 [70] and decreased UVB-induced apoptotic pathway in transgenic mice [71] probably as a strategy to evade oxidative stress-induced cell death and to promote long-term cellular survival in cooperation with the antiapoptotic protein Bcl-2 [72].

## 5. Limitations and Strengths of the Study

There are some limitations of our study that should be recognized:

- We did not provide in vivo data in support of our findings in HaCaT cells, and no in vitro model system can perfectly recapitulate the in vivo complexity of a disease in terms of immunological response and microenvironmental interactions
- We used a single cell line; however, HaCaT cells are widely used to study keratinocytes transformation upon UVA and UVB radiation [73] and are considered a valuable tool to study skin tumor promotion [33, 37]. In fact, despite being nontumorigenic [74] and approximating normal keratinocytes, HaCaT cells harbor UV-type p53 mutations [75] and thus are considered at an early stage of the multistep process of skin carcinogenesis [76].
- We used only UVA rays; despite representing the vast majority of UV received on Earth, the combination of UVA and UVB better simulates the solar radiation. However, no significant increase in the number of UVB-induced skin tumors in mice treated with HCTZ was reported [57].

The strengths of the study, making it translationally relevant, are as follows:

- We tested HCTZ in a range of concentrations reflecting the plasma levels of doses used in clinical practice [25, 26]; despite the lack of data on the accumulation of HCTZ in the skin, it is reasonable that HCTZ reaches the skin in a sufficient amount since patients taking HCTZ often experience cutaneous photosensitivity reactions; the use of therapeutic concentrations is also relevant for mimicking the actual human exposure.
- We applied a cumulative weekly UVA dose of 20 J/cm^2^ that mimics a human exposure of approximately 1 hour to midsummer sun in Paris [27], and it is below the UVA minimal erythema dose for I-II skin phototypes [77]; moreover, the long-term treatment resembles the multiple irradiations and chronic HCTZ treatment that may drive the cancerogenic process from cells able to survive upon insults and transform.
- The morphological and molecular features described resemble some aspects of dysplastic lesions described by Coussens and Hanahan [78] in a mouse model of squamous carcinoma and, at least in part, those found in precursor lesions and in nonmelanoma skin cancers in humans.

## 6. Conclusions

Our results demonstrate that the combined chronic exposure to HCTZ and UVA radiation is more prophotocarcinogenic than it would be expected from UVA alone and highlight the relevance of associating drug safety data with experimental evaluations to clarify the molecular mechanisms underlining adverse drug effects. In fact, HCTZ alone does not possess any direct damaging effect but rather potentiates that of UVA, in accordance with its photosensitizing properties. This further supports the recommendation made by the regulatory agencies for an accurate photoprotection especially while taking HCTZ.

## Data Availability

All data used to support the findings of this study are included within the article.
